# M13 phage-based antigen presentation and immune response activation in vaccine development for animal infectious diseases

**DOI:** 10.3389/fimmu.2026.1882961

**Published:** 2026-07-15

**Authors:** Guodong Zhou, Jiahui Zhang, Zhihui Zhang, Shengliang Cao, Yubao Li

**Affiliations:** 1 College of Agriculture and Biology, Liaocheng University, Liaocheng, Shandong, China; 2 Shandong Key Laboratory of Applied Technology for Protein and Peptide Drugs, Liaocheng University, Liaocheng, Shandong, China; 3 College of Pharmaceutical Sciences and Food Engineering, Liaocheng University, Liaocheng, Shandong, China

**Keywords:** infectious diseases, M13 bacteriophage, nanoparticles, surface display, vaccines

## Abstract

Phage display technology has emerged as a versatile platform for vaccine development by integrating antigen presentation with intrinsic immunostimulatory properties. Among the available phage systems, the filamentous bacteriophage M13 has attracted considerable attention because of its structural versatility, genetic programmability, and capacity for diverse antigen display strategies. Recent studies have demonstrated the potential of M13-based vaccines to induce robust humoral and cellular immune responses against a broad spectrum of animal viral, bacterial, and parasitic pathogens. This review critically summarizes current advances in M13 phage-based vaccine development, with particular emphasis on structural characteristics, antigen display strategies, immune mechanisms, and representative veterinary applications. In addition, we discuss key challenges associated with commercial translation, including scalable manufacturing, quality control, formulation stability, and future development. By integrating recent progress with remaining challenges, this review provides an updated perspective on the rational design and translational development of next-generation M13 phage-based veterinary vaccines.

## 1 Introduction

Animal infectious diseases represent a persistent and evolving challenge to animal health, livestock production, and food security worldwide (1). Despite long-standing control efforts, recurrent outbreaks of highly transmissible diseases, such as foot-and-mouth disease and avian influenza, continue to cause substantial economic and societal losses, reflecting the limited effectiveness of existing prevention strategies under real-world conditions (2–5). This challenge is further amplified by the continuous emergence of novel pathogens, antigenic variation among circulating strains, and the growing contribution of animal reservoirs to human infectious diseases (6–8). Together, these factors underscore the increasing complexity of infectious disease control at the animal–human interface and highlight the need for more adaptive and integrated prevention strategies, including the development of next-generation vaccines together with strengthened biosecurity and environmental control measures (9–12).

Vaccination remains the cornerstone of infectious disease prevention in veterinary medicine (13–15). However, the performance of conventional vaccine platforms has not kept pace with the increasing diversity and variability of animal pathogens (16, 17). Live attenuated and inactivated vaccines, while effective in certain contexts, are often associated with safety concerns, production constraints, or limited flexibility in responding to rapidly evolving pathogens (18). Recombinant subunit vaccines offer improved safety profiles but frequently suffer from insufficient immunogenicity and narrow protective breadth, particularly against antigenically heterogeneous or emerging pathogens (19, 20). These intrinsic limitations reveal a fundamental gap between traditional vaccine design paradigms and the immunological demands posed by contemporary animal infectious diseases. Overcoming this gap increasingly requires interdisciplinary innovation that integrates biomedical science with engineering, intelligent technologies, management strategies, and human-centered approaches to accelerate vaccine design, development, and practical implementation (21–25).

In this context, virus-based nanostructures have attracted increasing attention because they enable precise control over antigen organization while retaining intrinsic biological functionality (26, 27). Within this broad class of platforms, bacteriophages represent a particularly compelling option, as their viral architecture can be directly repurposed for vaccine construction (28). The feasibility of this approach was fundamentally enabled by the development of phage display technology, which allows foreign peptides or proteins to be genetically fused to phage coat proteins and repetitively presented on the phage surface (29). Through this strategy, bacteriophages were transformed from simple biological entities into modular and programmable antigen display scaffolds, thereby establishing a direct and controllable link between antigen structure and immune recognition (30). Notably, filamentous bacteriophages such as M13 possess structural features and stability profiles that align particularly well with the immunological demands identified for next-generation animal vaccines (31).

The filamentous bacteriophage M13 belongs to the family Inoviridae and exhibits a highly elongated morphology with an approximate length of 900 nm and a diameter of 7 nm (31). Its capsid is composed of approximately 2700 copies of the major coat protein pVIII arranged in a helical manner to form a tubular structure that encapsulates a 6.4 kb single stranded DNA genome (31, 32). The two ends of the virion are capped by minor coat proteins pIII, pVI, pVII, and pIX, which together define the structural polarity and functional organization of the phage particle (32). Unlike lytic bacteriophages, filamentous phages such as M13 establish a non-lytic infection cycle that allows continuous phage production without host cell destruction (31, 33). This biological feature enables rapid and scalable phage amplification, providing a practical advantage for vaccine development, particularly in scenarios requiring timely responses to emerging or evolving pathogens. Collectively, the well-defined architecture, host specificity, and production efficiency of M13 form the structural and biological foundation for its use as a programmable vaccine platform.

This review summarizes the current progress in the development of M13 bacteriophage-based vaccine platforms. Although M13-derived systems have not yet achieved widespread clinical implementation, existing studies clearly demonstrate their potential as safe, programmable, and versatile carriers for antigen display. By examining the structural features of M13, representative antigen display strategies, and their applications in animal vaccine development, this review provides critical insights and identifies key directions for improving immunogenicity, scalability, and translational feasibility in future veterinary vaccine research.

## 2 Structural and functional properties of the M13 bacteriophage

Bacteriophages constitute a diverse group of viruses that exclusively infect bacterial hosts and are commonly categorized according to virion morphology into icosahedral, tailed, and filamentous forms (34, 35). Filamentous bacteriophages are distinguished from lytic phages by their non-destructive replication strategy and by genomes composed of circular single stranded DNA. Members of this group primarily target gram negative bacteria harboring the F plasmid, which confers susceptibility to infection through the formation of F pili (31).

**Figure 1:**
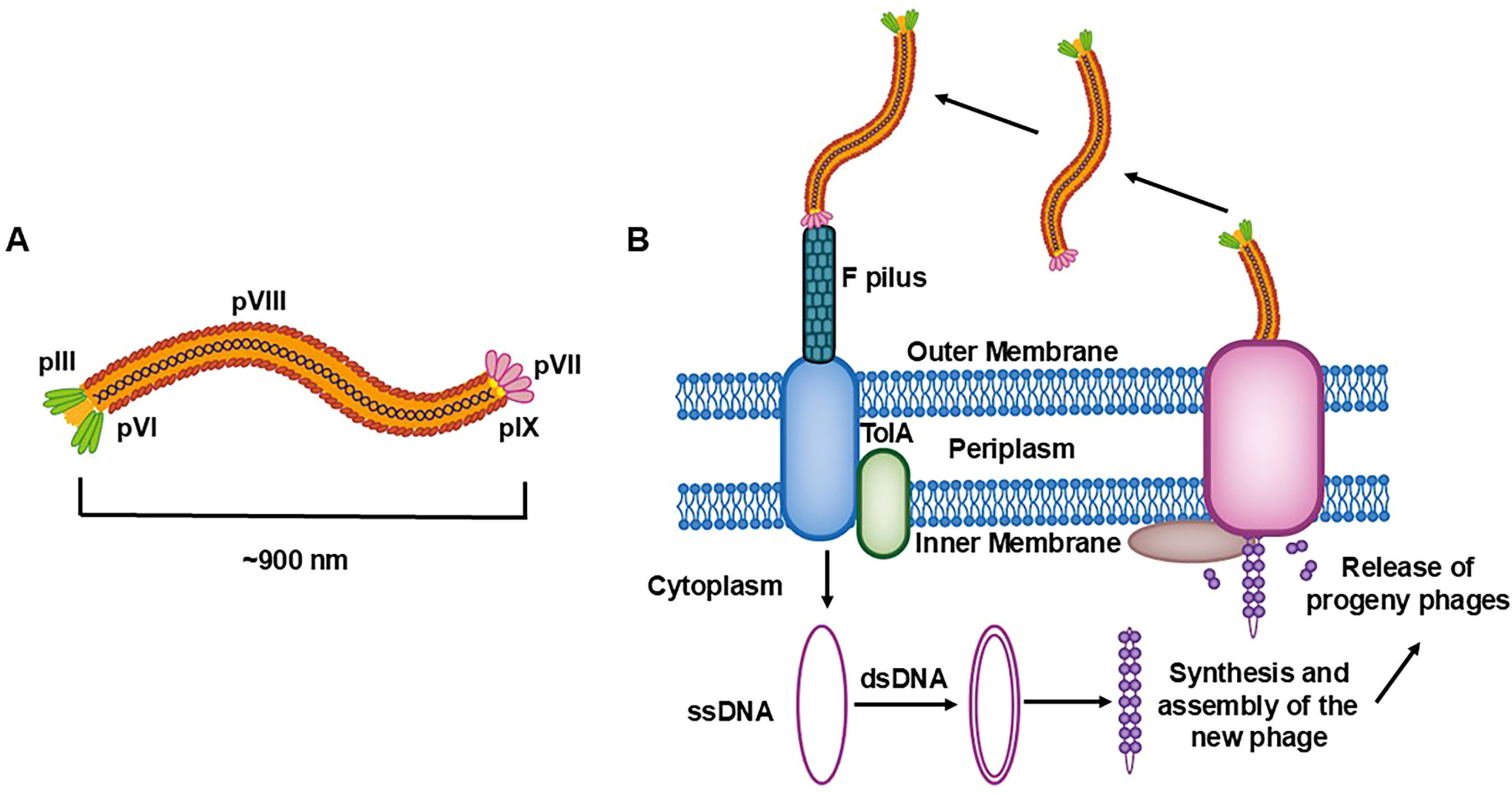
Structure and life cycle of the M13 bacteriophage. **(A)** Schematic representation of the M13 bacteriophage structure, showing its associated structural proteins (pIII, pVI, pVII, pVIII, pIX) along the length of the phage (~900 nm). **(B)** Overview of the M13 bacteriophage infection process. The phage attaches to the host cell through the F pilus. The phage DNA enters the bacterial cell via the TolQRA complex in the outer membrane, where the double-stranded DNA (dsDNA) is converted into single-stranded DNA (ssDNA). The ssDNA then undergoes replication, synthesis, and assembly of new phage particles, which are eventually released from the host cell to infect additional bacteria.

M13 bacteriophage is a representative filamentous phage belonging to the Ff group, together with closely related phages such as fd and f1 (36). The M13 virion exhibits a flexible, filament-like architecture with a diameter of approximately 6 to 7 nm and a length approaching 900 nm, as shown in **Figure 1A** (37). Its genome consists of a circular single-stranded DNA molecule of approximately 6.4 kb that encodes eleven proteins, designated pI to pXI (38). These proteins can be functionally categorized into capsid components, replication-associated proteins, and assembly-related proteins (39–41). Specifically, pIII, pVI, pVII, pVIII, and pIX constitute the structural framework of the virion, whereas pII, pV, and pX are primarily involved in genome replication, and pI, pIV, and pXI participate in phage assembly and secretion (as shown in **Figure 1A**) (42).

The major coat protein pVIII forms the backbone of the M13 capsid and is present in approximately 2700 copies, accounting for nearly 87% of the total phage mass, as shown in **Figure 1A** (42–44). Each pVIII subunit is composed of 50 amino acids and contains a hydrophilic N-terminal region, a central hydrophobic segment, and a positively charged C-terminal domain that interacts electrostatically with the viral genome (43). The tight and repetitive helical arrangement of pVIII around the single-stranded DNA generates a mechanically stable yet flexible filamentous scaffold, a feature that underlies the exceptional tolerance of M13 to extensive surface modification (43). In contrast, the minor coat proteins are asymmetrically distributed at the two ends of the virion, conferring polarity and functional specialization. Five copies each of pIII and pVI form the infective end of the phage, whereas five copies each of pVII and pIX cap the opposite terminus and participate in genome recognition and packaging.

Infection of the host bacterium is initiated through a multistep process mediated by the minor coat protein pIII, which is a 406-amino-acid protein comprising N1, N2, and C domains separated by glycine-rich linker regions, as shown in **Figure 1B** (43, 45, 46). Adsorption begins when the N2 domain of pIII binds to the distal tip of the F pilus, triggering conformational changes that expose the N1 domain (**Figure 1B**) (43, 47). The N1 domain subsequently interacts with the TolQRA complex embedded in the cytoplasmic membrane, facilitating genome entry. During this process, pVIII subunits are progressively stripped from the virion and integrated into the bacterial inner membrane, while the viral single-stranded DNA is translocated into the cytoplasm.

Once inside the host cell, the incoming single-stranded DNA is converted into a double-stranded replicative form by host DNA polymerases (43, 48). Replication proceeds via a rolling-circle mechanism initiated by the phage-encoded protein pII, which introduces a site-specific nick in the replicative form to generate new positive-strand genomes (49). The single-stranded DNA-binding protein pV accumulates during infection and binds newly synthesized genomes, preventing their conversion back into the replicative form and thereby directing them toward encapsidation (43, 50). In parallel, the protein pX modulates positive-strand synthesis by regulating pII activity, ensuring balanced genome replication (51). Assembly of new virions occurs at the bacterial membranes, where pI and pXI in the cytoplasmic membrane, together with pIV in the outer membrane, form a trans-envelope secretion channel (52). During assembly, the DNA packaging signal interacts with the C-terminal residues of pVII and pIX, initiating encapsidation (53). The pV protein is subsequently displaced by pVIII as the genome is extruded through the secretion channel, and incorporation of pIII and pVI at the distal end completes virion maturation (54). Notably, this entire process occurs without host cell lysis, allowing continuous phage production.

The combination of a precisely defined capsid architecture, a genetically tractable genome, and a non-lytic propagation strategy has established M13 bacteriophage as a highly adaptable biological scaffold. Its capsid proteins tolerate extensive genetic and chemical modification without compromising particle integrity, enabling fine control over surface composition and molecular presentation. These properties have facilitated the widespread use of M13 in phage display technologies, functional nanomaterial assembly, and targeted delivery systems. Collectively, the structural simplicity, biological robustness, and engineering flexibility of M13 provide a solid mechanistic foundation for its application as a programmable platform in vaccine development and related biomedical fields.

## 3 Antigen display strategies using the M13 bacteriophage

The structural organization of M13 directly underpins its antigen display capability and provides the basis for both conventional genetic fusion and emerging modular conjugation strategies (42, 44, 55). The distinct distribution and copy numbers of individual coat proteins enable diverse antigen display formats that can be tailored to different antigen sizes and immunological requirements (44, 55). In particular, pIII and pVIII have become the principal scaffolds for vaccine engineering, whereas recent SpyTag/SpyCatcher-mediated conjugation strategies have further expanded M13 engineering by enabling covalent antigen attachment without direct coat protein fusion (**Figure 1A**) (56).

**Figure 2:**
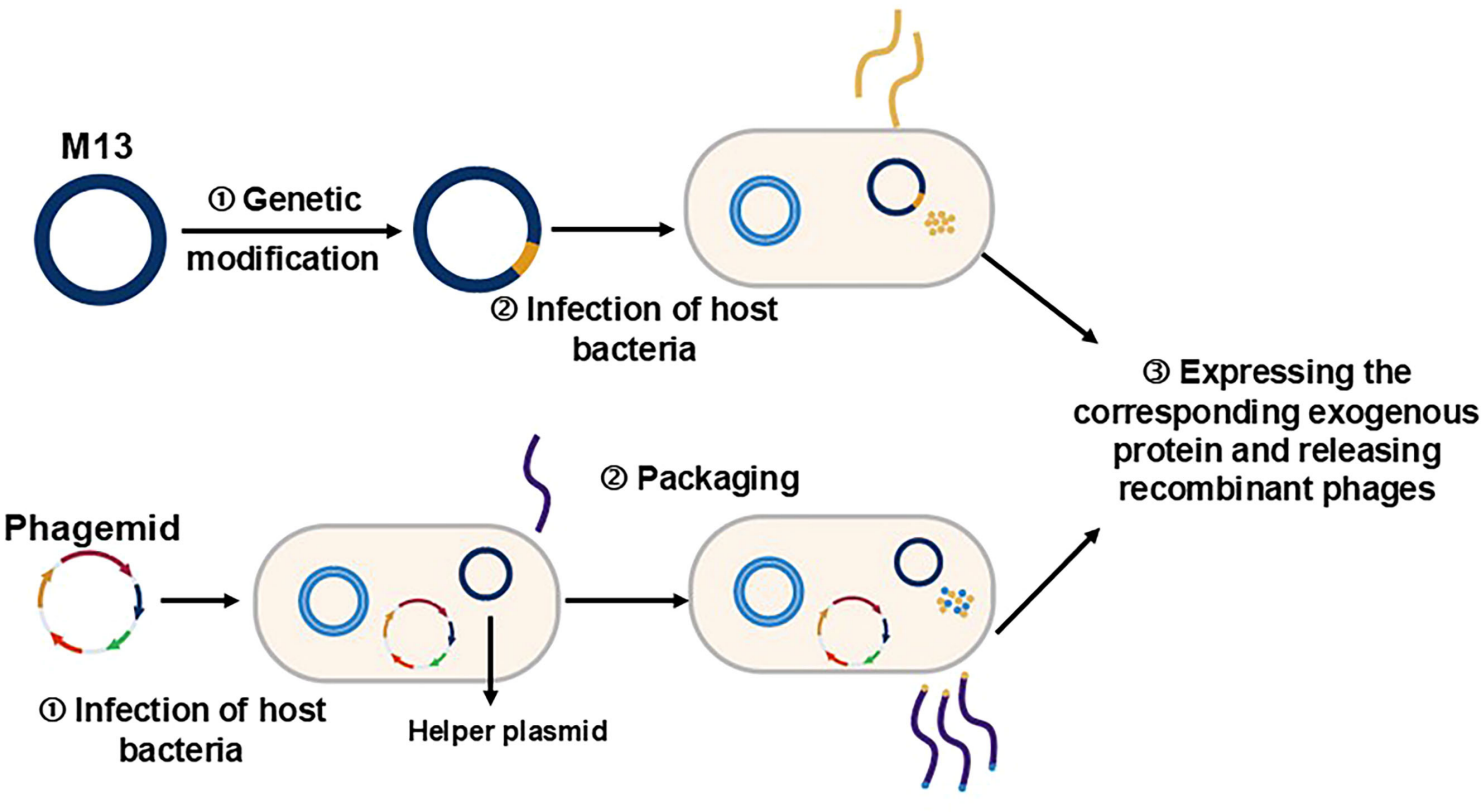
M13 based antigen display systems. Genome based display involves genetically fusing antigens to the coat proteins pIII or pVIII, while phagemid based display involves expressing recombinant coat proteins from a hybrid plasmid and assembling them with the help of a co-infecting helper phage.

Within this framework, conventional genetic fusion remains the most established strategy for M13 antigen display. Foreign peptides or proteins are fused to selected coat proteins, mainly pIII or pVIII, and are incorporated into phage particles during virion assembly. Depending on vector architecture and gene organization, antigen display can be achieved through genome-based vectors or phagemid-based systems, which differ in display valency, insert tolerance, and practical applicability, as illustrated in **Figure 2** (32). Phage genome-based display relies on engineered double-stranded replicative forms of the M13 genome, enabling efficient cloning, mutagenesis, and sequence verification prior to phage production (44). Recombinant phage genomes subsequently direct virion assembly while displaying foreign peptides or proteins fused to selected coat proteins. This strategy has been widely used to generate either uniform or mosaic display formats on pIII or pVIII, depending on whether wild-type coat protein genes are retained. In contrast, phagemid-based systems employ plasmid-phage hybrid vectors that require helper phages to provide structural proteins for virion assembly (57). Competition between recombinant fusion proteins and helper phage-derived wild-type coat proteins typically results in monovalent or low-valency display, facilitating the presentation of larger inserts and the construction of highly diverse libraries (58–60).

Emerging modular conjugation strategies have further broadened the engineering flexibility of M13 antigen display beyond conventional genetic fusion. Among these, the *Streptococcus pyogenes* FbaB-derived SpyTag/SpyCatcher system provides a versatile platform for site-specific protein conjugation (56, 61). SpyTag, a 13-amino-acid peptide, spontaneously forms an irreversible isopeptide bond with the 116-amino-acid SpyCatcher through a reaction between an aspartate residue in SpyTag and a lysine residue in SpyCatcher, enabling rapid and covalent antigen attachment under physiological conditions (61). Integration of this strategy with M13 engineering decouples antigen loading from phage assembly, thereby improving compatibility with structurally complex proteins while preserving display flexibility. More recently, programmable M13 platforms have combined SpyTag/SpyCatcher-mediated conjugation with rational control of antigen density, particle architecture, intrinsic adjuvanticity, and immune-targeting ligands, enabling coordinated optimization of antigen presentation and immune activation (56, 62, 63). Collectively, these advances establish M13 as a programmable vaccine engineering platform rather than a conventional antigen display scaffold.

## 4 Immune responses supporting M13 bacteriophage-based vaccination

M13 bacteriophage is not merely a passive antigen carrier but an immunologically active platform with intrinsic adjuvant properties. Its immunostimulatory activity is mainly associated with phage-derived pathogen-associated molecular patterns, including unmethylated CpG motifs within the single-stranded DNA genome and bacterial components that may be associated with phage preparations (64, 65). These signals can engage pattern-recognition receptor pathways and activate innate immune responses that shape subsequent adaptive immunity.

**Figure 3:**
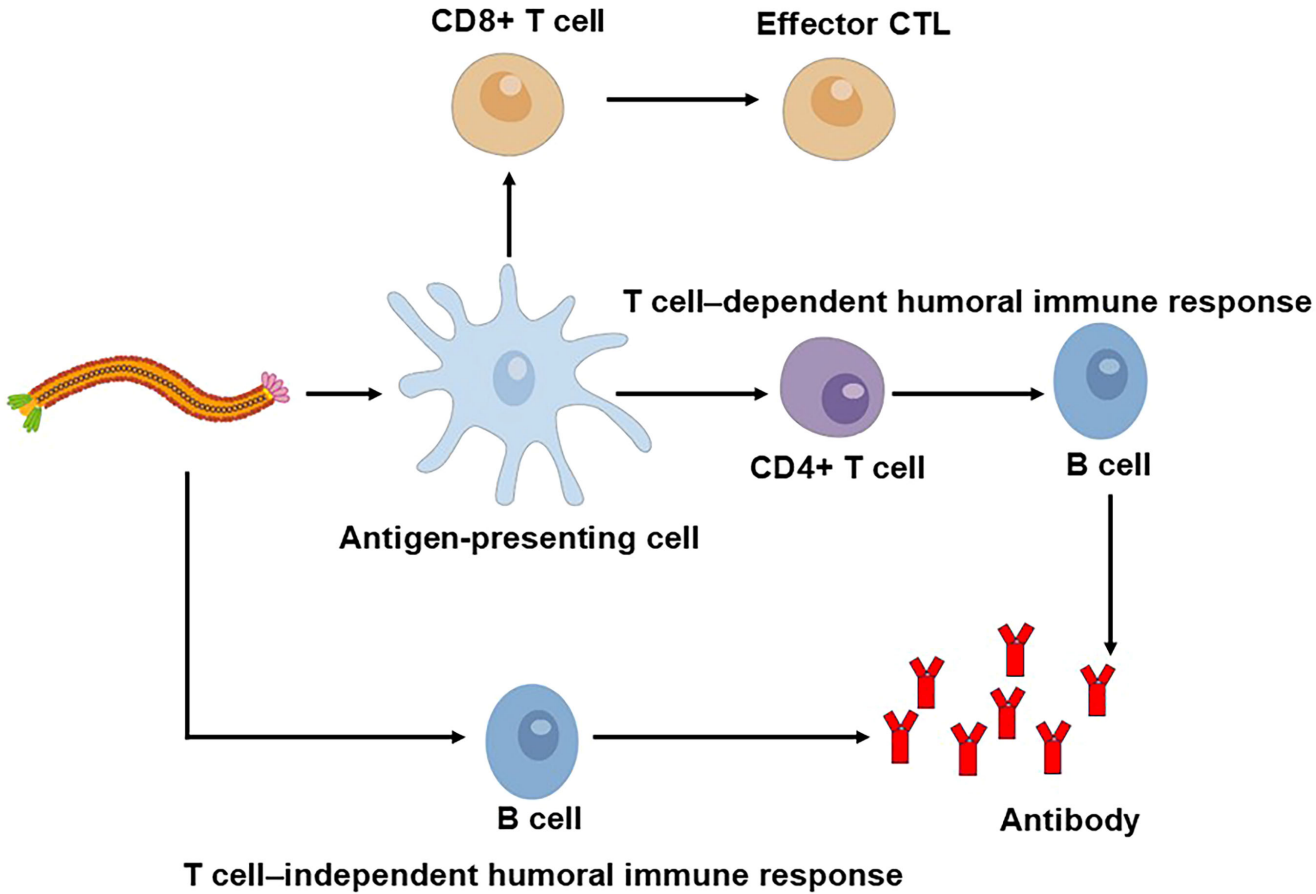
Immune responses supporting M13 bacteriophage-based vaccination. The figure illustrates the key immune pathways activated by M13 bacteriophage during vaccination. The phage induces both T cell-dependent and T cell-independent humoral immune responses. In the T cell-dependent pathway, antigen-presenting cells process phage antigens and present them to CD4^+^ T cells, leading to B cell activation and antibody production. The T cell-independent pathway involves direct activation of B cells, resulting in antibody secretion. Additionally, M13 phage triggers CD8^+^ T cell responses through cross-presentation of antigens, promoting effector cytotoxic T lymphocyte (CTL) activation.

These innate immune events provide the foundation for the induction of robust and long-lasting humoral immune responses by M13 bacteriophage (**Figure 3**) (66). After repeated immunization, anti-phage antibody titers can reach high levels and remain detectable for extended periods, indicating durable immunological memory (67). These antibody responses involve both T cell-dependent and T cell-independent B cell activation pathways (65, 68). The highly ordered and repetitive arrangement of pVIII on the M13 surface can efficiently cross-link B cell receptors, thereby promoting marginal zone B cell activation and antibody production (69). In addition, the shift from primary IgM and IgG2b responses to secondary IgG1 and IgG2b responses suggests the development of memory-associated humoral immunity and coordinated engagement of helper T cell-related pathways.

In parallel with humoral immunity, M13 bacteriophage also promotes potent cellular immune responses (**Figure 3**) (70–72). After uptake by antigen-presenting cells, phage-associated antigens can be processed and presented through MHC class II pathways to CD4^+^ T helper cells, leading to the activation of Th1, Th2, and Th17 subsets (73, 74). These helper T cells provide cytokine signals and support B cell differentiation, thereby linking cellular immunity with antibody production. Importantly, M13-associated antigens can also enter the MHC class I pathway through cross-presentation, resulting in the activation of CD8^+^ cytotoxic T lymphocytes and the generation of memory CTL responses (70, 71, 75). This ability to coordinate antigen presentation, helper T cell activation, and cytotoxic T cell priming is particularly relevant for vaccines against intracellular pathogens and tumor-associated antigens. Notably, the N1 and N2 domains of pIII contribute not only to phage infectivity but also to immune activation, as disruption of these domains markedly reduces phage uptake and attenuates immunogenicity (76).

Collectively, these findings demonstrate that M13 bacteriophage orchestrates innate, humoral, and cellular immunity through an integrated immunological mechanism. Its repetitive capsid architecture supports B cell receptor engagement, its genome and associated microbial signals provide innate immune stimulation, and its uptake by antigen-presenting cells enables both helper T cell activation and cross-presentation. These properties explain why M13 can function as a self-adjuvanting vaccine platform rather than a simple antigen display scaffold. Building on these mechanistic insights, the following section highlights representative applications of M13-based display systems in animal vaccine development and illustrates how these immune properties translate into protective vaccine efficacy.

## 5 M13 phage as a vaccine platform for animal viral diseases

A defining advantage of filamentous phage-based vaccines lies in their compatibility with diverse classes of viral antigens, including highly variable surface glycoproteins, conserved subdominant epitopes, and rationally designed multiepitope constructs (77–81). This versatility has been exemplified in vaccine strategies targeting antigenically unstable viruses such as influenza. Recombinant M13 phages encoding full-length hemagglutinin (HA) function as self-adjuvanting immunogens due to their CpG-rich genomes, which promote innate immune activation (77). When combined with inorganic nanomaterials such as MnO_2_ nanoparticles, these systems further overcome intracellular delivery barriers by facilitating endosomal escape and activating cytosolic DNA-sensing pathways, notably cGAS-STING, thereby amplifying dendritic cell maturation and T-cell priming. Complementary approaches exploiting the conserved influenza M2 ectodomain (M2e) or multiepitope constructs generated via reverse vaccinology illustrate how filamentous phage platforms can accommodate both strain-specific and broadly protective antigenic designs within the same structural framework (78, 79).

Beyond respiratory viruses, the capacity of M13 phages to support mucosal immunization has positioned them as promising candidates for enteric viral vaccines. In the case of porcine epidemic diarrhea virus, phage display of linear B-cell epitopes derived from the spike protein, coupled with epithelial-targeting peptides, enables efficient antigen delivery at mucosal surfaces (80). Such strategies elicit robust humoral and cellular immune responses following intranasal or oral administration, underscoring the suitability of filamentous phages for pathogens that require localized immune protection. Importantly, these examples demonstrate that phage-based vaccines can be rationally engineered to integrate antigen selection with tissue-specific targeting, a key requirement for effective mucosal immunity.

Notably, the applicability of filamentous phage vaccines is not restricted to mammalian systems. In aquaculture, where conventional vaccination strategies are often impractical, phage-based antigen display offers a low-cost and scalable alternative. Display of the white spot syndrome virus envelope protein VP28 on M13 phage confers protective effects against viral challenge in shrimp, reducing mortality without disrupting basal immune homeostasis (81). This cross-species applicability highlights a unique strength of phage-based platforms: their ability to function across distinct immune architectures while maintaining a consistent design logic.

## 6 M13 phage as a vaccine platform for animal bacterial diseases

In addition to viral infections, M13 phage–based vaccine platforms have also been investigated for bacterial diseases of veterinary importance, particularly those affecting livestock health and production efficiency (82). Bacterial pathogens remain a major cause of morbidity and mortality in young animals, and existing vaccines often provide incomplete protection or are associated with high production costs (83, 84). The genetic programmability and inherent immunogenicity of M13 phage make it an attractive scaffold for the development of novel veterinary vaccines.

A representative example is enterotoxigenic *Escherichia coli* (ETEC), a major etiological agent of neonatal diarrhea in calves (85). The pathogenicity of bovine ETEC is closely linked to fimbrial adhesins that mediate bacterial attachment to intestinal epithelial cells, among which the F17a fimbrial tip adhesin F17a-G plays a critical role in host colonization (86). To target this adhesion process, the receptor-binding domain of F17a-G has been genetically fused to the minor coat protein pIII of M13 phage, enabling its surface display in a functionally active form (82). The resulting recombinant phage retained specific carbohydrate-binding activity and induced antigen-specific IgG responses following immunization, demonstrating that M13 phage can effectively present bacterial adhesion domains while preserving immunogenicity.Although the antibody responses elicited by the phage-displayed F17a-G were moderate and did not fully block receptor engagement, this strategy provides a proof of concept for anti-adhesion vaccine design against ETEC in livestock.

Importantly, these findings highlight the feasibility of using M13 phage to deliver structurally and functionally intact bacterial antigens relevant to veterinary pathogens. Further optimization of antigen density, immunization route, and formulation, as well as validation in target animal species such as calves, will be essential to assess the translational potential of this approach.

## 7 M13 phage as a vaccine platform for animal parasitic diseases

Parasitic diseases continue to impose substantial burdens on animal health and livestock production, particularly in developing regions where control measures are difficult to implement and vaccination remains limited (87). The need for low-cost, scalable, and field-deployable vaccines has driven interest in alternative antigen delivery platforms. In this context, M13 phage has been explored not only as a vaccine carrier but also as a discovery tool for parasite antigens relevant to veterinary medicine (88, 89).

A prominent example is porcine cysticercosis caused by Taenia solium, a parasitic disease that adversely affects pig health and growth performance while also serving as a major source of human infection through the consumption of contaminated pork. To address the limitations of conventional control strategies, multiple protective antigenic peptides (KETc7, GK1, KETc1, and KETc12) were displayed on the surface of M13 phage to generate a multivalent vaccine formulation (88). Following sterilization, the resulting phage-based vaccine (S3Pvas-Phage) was evaluated under field conditions in endemic areas of Mexico. In a large-scale trial involving over one thousand pigs, vaccination significantly reduced infection prevalence and parasite burden without negatively affecting weight gain. These results demonstrate that M13 phage–based vaccines can achieve meaningful protection against parasitic infections under real-world production settings, highlighting their suitability for large-scale veterinary application in resource-limited regions.

Beyond vaccine delivery, M13 phage display has also been employed as a powerful platform for antigen discovery in parasitic diseases where candidate targets remain poorly defined. This approach has been illustrated in studies of Toxoplasma gondii, an intracellular protozoan parasite of veterinary relevance and zoonotic concern (89). By constructing a high-complexity M13 phage display library from fragmented T. gondii genomic DNA, researchers were able to screen for parasite-derived peptides recognized by specific antibodies. This strategy led to the identification of a peptide epitope corresponding to the dense granule antigen GRA3, confirming its immunological relevance. The identification of GRA3 through phage display underscores the utility of M13 phage not only in vaccine development but also in uncovering novel diagnostic and immunogenic targets for parasitic diseases.

## 8 Manufacturing and commercial translation of M13 phage-based vaccines

The promising results achieved in animal viral, bacterial, and parasitic disease models demonstrate that M13 bacteriophage is a versatile vaccine platform. However, successful translation from experimental studies to practical veterinary vaccines depends not only on immunogenicity but also on scalable manufacturing and consistent product quality. Benefiting from its non-lytic life cycle, M13 is continuously secreted from F pilus-positive *E*. *coli*, making bacterial fermentation a practical approach for large-scale production. Nevertheless, commercial manufacturing still requires standardized upstream production, scalable purification strategies, and robust quality control to ensure batch-to-batch consistency.

As production moves from the laboratory to industrial scale, quality control becomes a key consideration. Unlike mammalian cell-derived vaccines, the primary manufacturing risks of M13-based vaccines originate from bacterial host-derived impurities, including endotoxins, host cell proteins, host genomic DNA, residual helper phage, and incomplete phage particles. Consequently, regulatory evaluation should focus on the control of these process-related impurities together with genetic stability and antigen-display consistency. Comprehensive characterization of identity, purity, potency, sterility, endotoxin levels, residual host cell DNA and proteins, and particle integrity is therefore essential to ensure product safety and reproducibility.

Product stability is another important consideration for the practical deployment of M13-based veterinary vaccines. Although filamentous M13 particles exhibit excellent structural robustness, the thermal stability of recombinant vaccines should be evaluated individually because displayed antigens, genetic modifications, formulation composition, and storage conditions may all influence particle integrity and immunogenicity. Therefore, formulation development should be considered an integral part of vaccine optimization rather than a final manufacturing step. Lyophilized or other dried formulations may improve thermal tolerance and reduce dependence on cold-chain transportation, whereas appropriate excipients can further enhance long-term stability during storage and distribution. Future studies should combine formulation optimization with systematic stability evaluation under different storage conditions to facilitate large-scale production and field application of M13-based veterinary vaccines.

## 9 Concluding remarks

M13 phage represents a versatile and cost-effective platform for veterinary vaccine development by integrating programmable antigen display, intrinsic immunostimulatory activity, and scalable bacterial production within a single filamentous nanoparticle (31). These properties enable efficient antigen presentation and coordinated activation of innate, humoral, and cellular immune responses, supporting broad applications against viral, bacterial, and parasitic diseases. Although further improvements in immune durability, antigen display strategies, formulation design, and manufacturing standardization are still required, continued advances in phage engineering are expected to further enhance its translational potential. These desirable characteristics are increasingly shared by other virus-derived nanoplatforms, highlighting a broader shift toward the exploitation of naturally occurring viral architectures for vaccine development. For example, plant virus-based nanoparticles have likewise demonstrated excellent structural stability, multivalent antigen display capacity, favorable biosafety profiles, and scalable production, underscoring the versatility of non-mammalian viral platforms as programmable vaccine scaffolds (90). Together, these complementary platforms provide a strong foundation for the rational development of next-generation vaccines, with M13 phage remaining one of the most promising candidates for advancing veterinary vaccinology.
